# A Curious Case of Common Peroneal Nerve Schwannoma

**DOI:** 10.7759/cureus.56427

**Published:** 2024-03-18

**Authors:** Manimaran Ramachandran, Aiswerya Shankar

**Affiliations:** 1 Plastic Surgery, Sree Balaji Medical College and Hospital, Chennai, IND; 2 General Surgery, Sree Balaji Medical College and Hospital, Chennai, IND

**Keywords:** neurinoma, schwannomatosis, verocay body, neurilemmoma, peroneal nerve schwannoma

## Abstract

Schwannoma or neurilemmoma is a slow-growing tumor that develops from nerve sheaths. It is mostly benign and only rarely transforms into malignancy. The incidence of schwannoma is very low in the lower limbs. Schwannomas developing from the common peroneal nerve is unlikely. A middle-aged male presented with complaints of left knee pain, which was radiating to the left foot, and a painful swelling at the back of the knee. An intralesional excision was done, and the patient made a full recovery with no postoperative complications. The excised specimen was found to be a schwannoma of the common peroneal nerve of the left leg. At the one-month, three-month, and one-year postoperative follow-ups, the patient had no complaints of pain on passive and active dorsiflexion of the foot. There was complete recovery from paresthesia and intact sensation was present. This report shows that asymptomatic schwannomas can sometimes present with symptoms of pain. In such cases, careful and complete excision of the schwannoma can lead to full recovery.

## Introduction

Schwannomas are benign tumours of the nerve sheath and come from differentiated Schwann cells. Schwannomas affect all ages equally but can be more commonly seen in the age of thirty to sixty years. It has no gender predilection and almost ninety percent occur sporadically [1]. Three percent of schwannomas occur with Neurofibromatosis (NF) Type 2 and in a small group of patients, it occurs with Schwannomatosis. In five percent of cases, the schwannomas occur with multiple meningiomas in various locations in the presence or absence of NF Type 2 [1].

Schwannomas of the common peroneal nerve are uncommon and hence very few in literature. In general, schwannomas are asymptomatic but in this case of common peroneal nerve schwannoma, the patient had symptoms of pain due to mechanical compression.

MRI is considered the gold standard investigation as it helps to identify the size and exact location of the tumour along with other features such as cystic degenerative changes, edema surrounding the tumour, and its infiltration into the muscular plane.

We present an unusual case of schwannoma arising from the common peroneal nerve. This report describes the clinical presentation of schwannoma along with its histology, surgical findings, and functional outcome.

## Case presentation

A fifty-year-old Asian male, known hypothyroid for eight years on regular treatment, came to the outpatient department with complaints of worsening left knee pain. He had a one-month history of intermittent left knee pain, which was radiating to his left foot. A painful swelling was observed at the back of the knee, worsened by touch and pressure.

**Figure 1:**
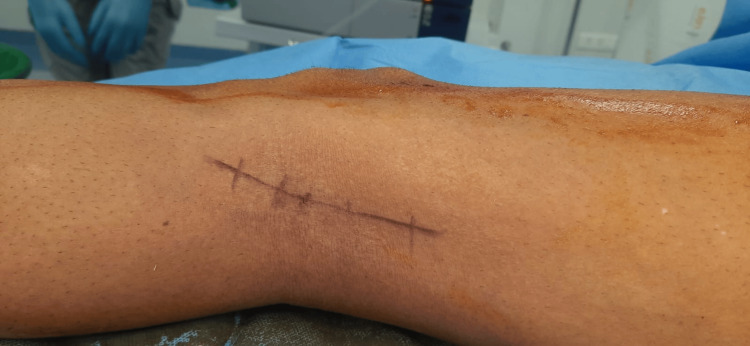
Swelling marked in the posterior aspect of left knee

He was not a smoker, not an alcoholic, and had no history of substance abuse. On examination, he was found to have a swelling of 1 to 1.5 centimeters in size in the posterolateral aspect of the left knee, firm in consistency. Severe tenderness was present that was aggravated on touch or pressure. Trans-illumination and fluctuation were negative. No other palpable swellings were present and there was no neurovascular deficit beyond the swelling (Figure 1).

**Figure 2:**
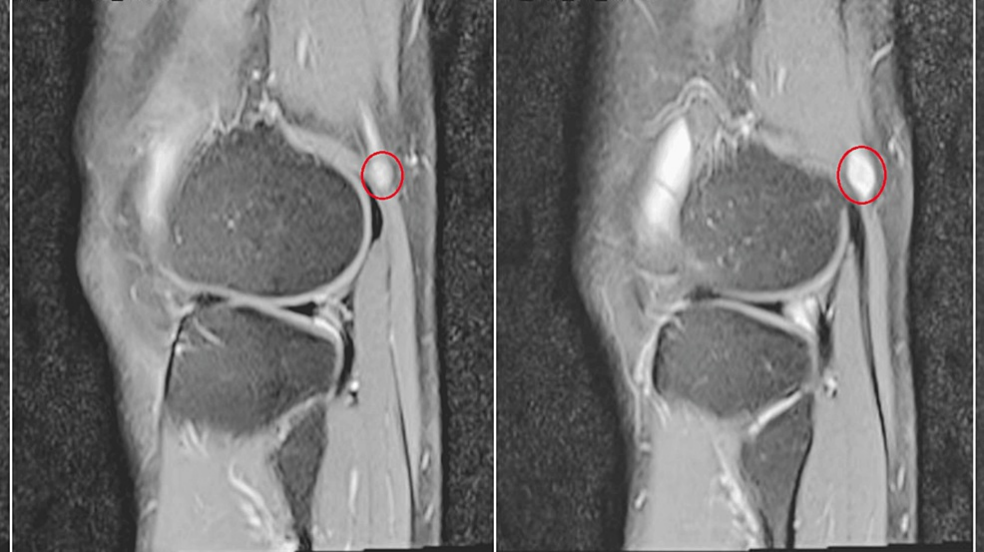
MRI showing an oval-shaped T1 Hypo/T2 hyperintense lesion(marked) in the posterolateral aspect of the left knee

**Figure 3:**
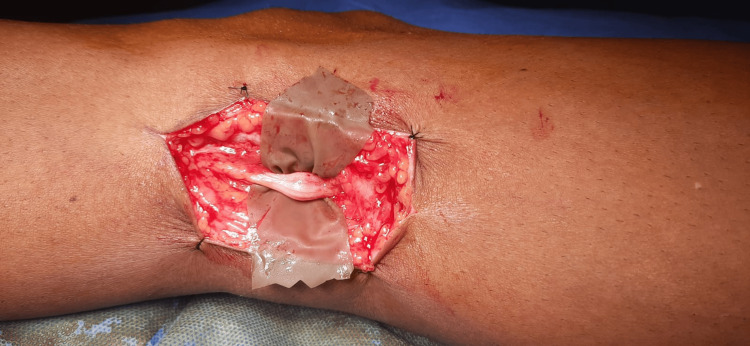
Intraoperative image of schwannoma

He experienced increased pain with passive and active dorsiflexion. While active foot inversion of the left foot, both with and without resistance, he experienced pain and discomfort. By mild tapping of the leg lump, there was paresthesia on the dorsum of the foot signifying that Tinel's sign is positive. He had a previous history of axillary nerve schwannoma- which was excised seven years ago. His blood investigations showed a hemoglobin count of 13.9g/dl, a total WBC count of 7,600, and a hematocrit value of 43%. His renal function tests and other laboratory investigations, including urine, results were unremarkable. MRI showed the possibility of peripheral nerve sheath tumor in the common fibular nerve and tibial nerve (Figure 2). The patient was referred to a plastic surgery specialist and underwent intralesional excision under spinal anesthesia (Figure 3). The excised material was sent to the histopathology laboratory.

 

**Figure 4:**
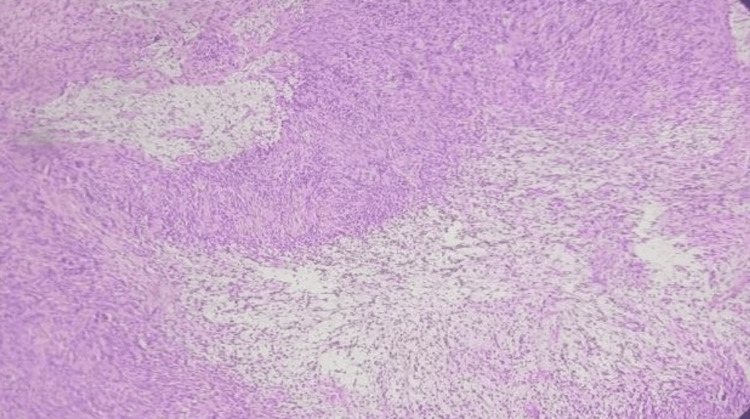
Histopathological image showing hypocellular areas with stromal oedema

**Figure 5:**
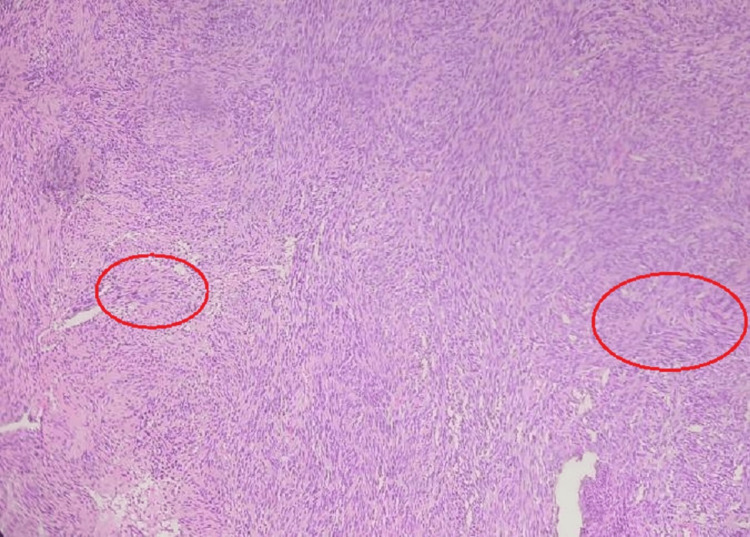
Histopathological image showing Verocay bodies (marked), consistent with schwannoma

A histopathological examination showed circumscribed neoplasm composed of cells arranged as sheets and ill-defined fascicles. Individual spindle-shaped cells (Antoni A) with Ill-defined borders with a moderate quantity of predominantly eosinophilic cytoplasm, round to oval nuclei with chromatin were also observed. Occasional hypocellular areas (Antoni B) with stromal edema (Figure 4), composed of cells with mildly vacuolated cytoplasm, were noted. Palisading of nuclei with Verocay bodies was also noted (Figure 5), consistent with schwannoma of the common peroneal nerve. Postoperatively, the patient's symptoms improved, and the patient made a full recovery. At the one-month follow-up, there was intact sensation of the left foot, complete recovery from paresthesia, and no evidence of foot drop. At the three-month and one-year postoperative follow-up visit, the scar was healthy, the patient had no complaints of pain on passive and active dorsiflexion of the foot, and range of movement at the knee joint was full and free.

## Discussion

Schwannomas are slow-growing tumors that develop from nerve sheaths, and hence, are generally painless and asymptomatic. This case report discusses a painful schwannoma arising from the common peroneal nerve. They come from differentiated Schwann cells. Schwannomas can be one or many in number. Multiple schwannomas can be seen in patients who also have been diagnosed with conditions such as NF Type 2 and Carneys complex or schwannomatosis [2]. The principal cause of familial tumor syndromes is the dysfunction or mutation of the tumor suppressor gene, *merlin* (*schwannomin*). Schwannomas are different from neurofibromas in the histopathological aspect. Neurofibromas are made of mast cells and axons of nerve cells whereas schwannomas are made of Schwann cells. Unlike neurofibromas, schwannomas develop from and are confined to the sheath and lie on top of the nerve. They do not traverse through the nerve. The risk of metastasis is less than one percent. Ancient schwannomas are long-standing schwannomas that have additional features of cystic hemorrhagic changes and degenerative nuclei with pleomorphism and hyperchromasia.

The most common region of presentation of neurilemmomas is the upper limbs. They are also seen in the oral cavity, orbit, and salivary glands. Tumors in the deep plane are seen mostly in the mediastinum and retroperitoneum. These tumors are rarely seen in the penis and vulva [3]. In 1908, these tumors were considered the most common benign peripheral nerve sheath tumors. However, schwannomas arising from the common peroneal nerve are notably very few in the literature [4,5].

The muscles of dorsiflexion and inversion of the foot are innervated by a deep peroneal nerve (a branch of the common peroneal nerve). Another component of the common peroneal nerve is the superficial peroneal nerve. This is the nerve supply of all muscles that perform eversion and plantar flexion. Owing to its unique superficial course around the posterolateral aspect of the head and neck of the fibula, traumatic injuries are far more common than non-traumatic lesions. During surgical excision, peroneal nerve injury should be avoided as it may lead to a common complication of foot drop [6,7].

## Conclusions

Schwannomas found in the proximal aspect of the lower limbs may cause distal symptoms. Although generally asymptomatic, once neurilemmomas become symptomatic, the treatment of choice is excision and careful follow-up. Nerve or soft tissue damage can be eliminated by thorough clinical examination, prompt investigation, and timely diagnostic confirmation.
